# Editorial: Reviews in psychiatry 2022: aging psychiatry

**DOI:** 10.3389/fpsyt.2023.1217186

**Published:** 2023-05-25

**Authors:** Shaheen E. Lakhan

**Affiliations:** Department of Biosciences, Global Neuroscience Initiative Foundation, Boston, MA, United States

**Keywords:** depression, Alzheimer's disease, microbiota, aging, geriatric psychiatry

Aging psychiatry is a subfield of psychiatry that focuses on the mental health needs of older adults, including those aged 65 and above. This field encompasses a wide range of mental health issues that are common in older adults, including depression, anxiety, cognitive decline, dementia, delirium, and substance abuse. These conditions can have a significant impact on the quality of life of older adults and their families, and often require specialized assessment and treatment approaches.

Our Research Topic “Reviews in Psychiatry 2022: Aging Psychiatry” focuses on on two main issues: (1) depression and (2) neurodegenerative disorders including Alzheimer's disease.

Perimenopause is characterized by fluctuations in hormonal levels, particularly estrogen and progesterone, which can lead to a range of physical and emotional symptoms, including mood changes and depression. Perimenopausal depression is a relatively understudied area compared to other aspects of menopause and women's health. Gao et al. did a bibliometric analysis and determined that the United States published the most articles on the subject followed by China, and the leading institution is Harvard University, and the leading publication Menopause: The Journal of the North American Menopause Society.

Many studies have reported high rates of depression among caregivers of cancer patients. Pan and Lin performed a meta-analysis and determined the weighted average prevalence of depression was 25.14%, less than those caring for stroke survivors and Alzheimer's disease (40.2 and 34.0%, respectively). They also found that geographical regions, caregiver characteristics, and patients' cancer stage factor the prevalence of depression. The high prevalence of depression among caregivers of cancer patients highlights the importance of providing support and resources to help caregivers cope with the emotional and practical challenges of caring for a loved one with cancer. Interventions such as psychotherapy, support groups, and respite care have been shown to be effective in reducing symptoms of depression and improving the quality of life for caregivers.

The association between microbiological risk factors and neurodegenerative disorders is an area of active research and ongoing investigation, and is the topic covered by Wang et al.. One of the most studied microbial risk factors for neurodegenerative disorders is the bacterium, Porphyromonas gingivalis (P. gingivalis), which is commonly associated with chronic periodontitis, a type of gum disease. Studies have shown that P. gingivalis can travel from the mouth to the brain, where it may trigger an immune response and cause neuroinflammation. In addition, P. gingivalis has been found to produce a toxic protein, called gingipain, which can damage brain cells and increase the accumulation of beta-amyloid, a hallmark feature of Alzheimer's disease.

Chen et al. report on the neuroprotective effects of irisin, a hormone that is produced in the body during exercise. There is some evidence to suggest that irisin may protect against Alzheimer's disease, a progressive neurodegenerative disorder that is characterized by the accumulation of amyloid-beta plaques and tau protein tangles in the brain. In addition to its effects on amyloid-beta, irisin may also protect against Alzheimer's disease by promoting the growth and survival of neurons in the brain. Neurodegeneration, or the loss of neurons, is a hallmark of Alzheimer's disease, and preserving the health and function of neurons is critical to preventing the cognitive decline associated with the disease. Studies have shown that irisin can increase the production of brain-derived neurotrophic factor (BDNF), a protein that is essential for the growth and survival of neurons in the brain.

Further research is needed in aging psychiatry because older adults are more likely to experience mental health problems, such as depression and cognitive impairment, and may face unique challenges in accessing and receiving appropriate treatment. There is a need for better understanding of the complex interplay between mental health and aging, as well as the specific mechanisms underlying these relationships. Additionally, there is a need for research on effective interventions for treating mental health problems in older adults.

## Author contributions

The author confirms being the sole contributor of this work and has approved it for publication.

